# The association between shift work disorder and turnover intention among nurses

**DOI:** 10.1186/s12912-022-00928-9

**Published:** 2022-06-06

**Authors:** Kjersti Marie Blytt, Bjørn Bjorvatn, Bente E. Moen, Ståle Pallesen, Anette Harris, Siri Waage

**Affiliations:** 1 Department of Health and Caring Sciences, Western Norway University of Applied Sciences, P.O. Box 7030, 5020 Bergen, Norway; 2 Department of Global Public Health and Primary Care, University of Bergen, Bergen, Norway; 3 Norwegian Competence Center for Sleep Disorders, Haukeland University Hospital, Bergen, Norway; 4 Department of Psychosocial Science, University of Bergen, Bergen, Norway; 5 Optentia, the Vaal Triangle Campus of the North-West University, Vanderbijlpark, South Africa

**Keywords:** Turnover intention, Shift work disorder, Nursing, Sleep

## Abstract

**Background:**

Shift work disorder (SWD) is highly prevalent among shift-working nurses and has multiple negative health-related effects. There is a dearth of insight into career-related decisions made by nurses suffering from SWD, for instance in terms of their intention to quit work (turnover intention). In this study, we aimed to investigate the association between SWD and turnover intention among nurses, and the individual and work-related correlates of turnover intention.

**Method:**

Data were derived from the ongoing longitudinal cohort study “SUrvey of Shift work, Sleep and Health (SUSSH)” among Norwegian nurses. An annual survey was initiated in 2008/2009 (*N* = 2965). The present study used data collected in year 2015 (wave 7) and 2016 (wave 8). Nurses were included if: 1) they were working as nurses in both 2015 and 2016, and 2) had completed a three-item scale adapted from the Michigan Organizational Assessment Questionnaire assessing turnover intention (in wave 8), and 3) did not only work day-shifts. SWD was measured in wave 7 with three questions based on the minimal criteria from the third edition of the International Classification of Sleep Disorders. Job demands, decision latitude, and social support at the workplace were measured with subscales of the Swedish Demand-Control-Support Questionnaire.

**Results:**

Eight Hundred eighty-nine nurses were included. The results from the hierarchical linear regression showed that SWD predicted turnover intention one year later, i.e. from 2015 to 2016 (F_1,835_ = 6.00, *p* < 0.05; β = 0.084, *p* = 0.015). The findings remained significant when controlling for age, sex, organizational tenure, number of nights worked, shift work schedule and workplace social support, job demands and decision latitude.

**Conclusion:**

This study showed that SWD is associated with turnover intention, even when controlling for individual and work-related variables.

## Introduction

In the European Union, as many as 40% of health care workers are exposed to shift work [1]. Shift work is associated with several negative health outcomes, such as impaired cognitive functioning [2], cardiovascular disease [3], ischemic stroke [4], depression [5], cancer [6, 7], metabolic syndrome [8], ulcers [9], obesity [10, 11], reproductive problems [12] and gastrointestinal dysfunction [9]. Furthermore, shift work is known to disrupt sleep and cause sleepiness [13]. Especially night shifts, early morning shifts and quick returns are known to cause significant sleep loss [14–16].

Shift work disorder (SWD) is associated with a recurring work schedule that overlaps with the usual time for sleep [17]. In 2014, a third edition of the International Classification of Sleep Disorder was published including amendments of the criteria of SWD. According to the ICSD-3 criteria for SWD, symptoms of insomnia/sleepiness should be linked to a reduction of total sleep time in relation to the work schedule. Furthermore, the required duration of the symptoms warranting a diagnosis increased from one (ICSD-2) to three months.

The prevalence of SWD varies by occupation and working time patterns. When SWD is measured by the criteria in ICSD-2, the prevalence is estimated to range from 10 to 84% [18–21]. A prevalence ranging from 29% in two-shift rotation workers to 44% in three-shift rotation workers was found among Norwegian nurses [22]. Few studies have investigated the prevalence of SWD using the criteria from ICSD-3. Still, a recent meta-analysis indicates that the ICSD-3 criteria yield somewhat lower prevalence estimates of SWD then the ICSD-2 criteria [23].

Beyond the health-related outcomes, SWD may also influence nurses’ career-related decisions, e.g., the decision to stay in or leave one’s job. Researchers have long investigated the relationship between working time arrangements and the intention to leave the job, both in the context of nursing [24, 25] and other professions [26]. There is however a dearth of knowledge about how SWD may influence turnover intention among nurses.

Turnover intention is defined as an employee’s intent to find a new job with another employer [27]. While turnover itself may not be problematic, excessive turnover may lead to reduced quality in clinical care and can be costly for health institutions [28]. Internal turnover (within the same organization) or external turnover (leaving the organization or the profession entirely) are both common among nurses [28]. Importantly, turnover intention does not necessarily lead to actual turnover, but has been shown to be a strong predictor for the latter [29].

Several studies have elucidated different factors that may lead to turnover intention among nurses. First, it has been shown that turnover intention is higher among younger versus older nurses [7, 30]. Previous research shows that age as well as organizational tenure (i.e., number of years working as a nurse) – two characteristics that may often correlate – are both positively related to organizational commitment and negatively related to turnover intention [31]. Other person- or role-related characteristics that have been found to be associated with turnover intention include sex [32], sleep disturbances [7, 33] working in frontline clinical roles and residential aged-care units [30] in addition to being less satisfied with pay or opportunities for career advancement [25].

A second strand of findings on the correlates of turnover intention relates to nurses’ experiences of their working environment. Parry et al. [30] found that turnover intention is higher among nurses who are less satisfied with their jobs and who often have experienced injury or abuse at the workplace in the preceding 12 months. Furthermore, work-family conflict (i.e., perception of insufficient energy and time to successfully perform work and family roles; cf. [34]) have been found to predict seeking of other job opportunities [35]. Finally, Yoon [25] found that nurses who work shifts and who desire daytime work also have higher turnover intention.

Turnover intention has multiple consequences both for the individual and for the society. Several theories have been developed in understanding the phenomenon of turnover intention. As reviewed and summarized by Holtom et al. ([36] p. 232) seven main trends have characterized the development of the understanding of turnover intention. These trends comprise (1) the role of individual difference as predictors of turnover (e.g., personality); (2) the role of stress- and change-related attitudes (e.g., change acceptance); (3) the role of unfolding decisions in several paths towards turnover (e.g., shocks and various behavioral responses); (4) the role of contextual variables and in particular interpersonal relationships (e.g., organizational diversity); (5) the role of factors that predict the inclination to stay in the job (e.g., job embeddedness); (6) the role of time and a process-orientation in the understanding of turnover (e.g., changes in employee attitudes over time), and finally, (7) the further investigation of previously identified relationships with turnover intention (e.g., meta-analyses). The multitude of factors covered here and the relative lack of integration between them, suggests relatively little theoretical consensus in the understanding of turnover intention.

Taken together, previous research shows that turnover intention depends on various individual and work-environmental factors. In this paper, the job demands-control-support (JDCS) model [37–39] functions as the underlying framework. It includes (1) job demands, i.e., psychological stressors related to work load and work conflicts [37, 40], (2) job control (or decision latitude), i.e. workers’ ability to control their work activities [40] and (3) social support at work, i.e., supportive interactions with co-workers [38, 41].

Previous research indicates that at the individual level, there are different stressors that affect people in different manners. According to the two-dimensional work stressor framework [42], challenge stressors could potentially promote personal growth, while hindrance stressors are potentially constraining the individual personal development and work-related accomplishment [43]. The meta-analysis conducted by Podsakoff et al. [42] found that hindrance stressors were negatively associated with job satisfaction and organizational commitment. As SWD is characterized by symptoms of insomnia/sleepiness, it might lead to adverse strain and impaired health, which in turn might negatively affect job satisfaction and organizational commitment.

Based on reported research, we therefore hypothesized that SWD is positively associated with turnover intention among nurses (i.e. nurses with SWD have higher degree of turnover intention). Furthermore, in light of previous research on shift work-environmental factors, we wanted to investigate whether this relationship remained when controlling for number of nights worked, shift work schedule, social support, job demand and decision latitude at the workplace. Finally, considering previous research on age, organizational tenure and sex, we wanted to investigate whether the relationship between SWD and turnover intention remained also when controlling for these individual factors.

## Methods and materials

### Procedure and participants

Data were derived from the ongoing longitudinal cohort study “SUrvey of Shift work, Sleep and Health (SUSSH)” among Norwegian nurses initiated in 2008/2009. In 2008, 6000 nurses were randomly selected from the Norwegian Nurses Organisation’s (NNO) membership roll and invited to participate in the study. Most Norwegian nurses have NNO membership. A total of 2059 nurses participated in the first wave (response rate = 38.1% when excluding 600 questionnaires that were returned due to wrong addresses). In 2009, an additional sample of 905 newly educated nurses were recruited (response rate = 33.1%) to increase the size of the study population. Thus, the total number of included nurses at baseline (2008/2009) was 2964. Of these, 1863 were available for inclusion in 2015.

All nurses included at baseline have since been invited to participate in annual follow-up surveys. Every year, the nurses who responded to the first wave received invitations to participate, including questionnaires by postal mail with prepaid return envelopes. Non-responders received up to two reminders. To increase participation rate, a lottery was conducted for each wave, in which 25 nurses could win a gift card with a value of 500 NOK (~ 50 US $).

### Inclusion criteria

The present study reports findings based on data collected in wave 7 (2015) and wave 8 (2016) and includes only persons who (1) were still working as nurses, (2) had a work schedule that was not limited to day-shifts only, and (3) who had completed the Michigan Organizational Assessment Questionnaire assessing turnover intention (TIS) in 2016, which is the dependent variable in the present analyses. Of the 1863 participants available for inclusion, 974 were excluded based on the criteria above. This provided a final sample of 889 participants.

### Demographical and work schedule variables

The demographic variables were all collected in 2015, except for sex and age, which were collected at baseline. However, the age variable was recalculated for the year 2015. In addition, we registered the following work-related variables in 2015: Number of nights shifts worked in the last year and number of years working as a nurse. Finally, we collected data on the nurses’ shift work schedules, for which we generated separate dummy variables: 1) evening shifts only, 2) night shifts only, 3) two-shift schedules (day and evening), 4) three-shift schedules (day, evening and night), 5) other schedules that included night work. The latter category served as the reference category in the coding of the dummy variables. The dummy variable for evening shifts only was excluded from the regression analyses, as there was only one person selecting this category. The shift-work schedule data was self-reported. The evening/night shift categories were largely similar for all participants and were defined as follows: Evening shifts typically begin at 14.30 and end at 22.00. Night shifts typically begin at 21.30 and end at 07.00. There is some slight variation, e.g., evening shifts that begin at 15.00 and night shifts that begin at 22.00; however, this variation is limited to such 30-min differences.

### Shift Work Disorder (SWD)

SWD was measured in 2015 with three questions based on the criteria from the third edition of the ISCD-3 (American Academy of Sleep Medicine 2014). The questions were formulated as follows: a) “Do you have a work schedule that sometimes overlaps with the time you usually sleep?”; b) “If yes, does this cause insomnia and/or excessive sleepiness due to reduced amount of sleep?”; c) “If yes, has this lasted for at least three months?”. If the nurses answered yes to all three questions, they were classified as having SWD. It is important that this classification of SWD versus non-SWD has not been validated against a clinical diagnosis. Our measure of SWD is thus only a proxy for SWD. Still, we henceforth refer to nurses who have responded yes to all three questions above as having SWD, which is in line with previous studies [19, 44].

### Turnover intention

Turnover intention was measured in 2016 with a three-item scale adapted from the Michigan Organizational Assessment Questionnaire [45]. The items were formulated as follows: 1) “I will actively look for a new job in the next year,” 2) “I often think about quitting” and 3) “I will probably look for a new job by the next year”. Responses were noted on a 5-point Likert scale from 1 (strongly disagree) to 5 (strongly agree). We added the scores of the three items into one composite score, which ranged from 3 to 15. A higher score reflects a higher degree of turnover intention. The scale had high internal consistency (α = 0.941).

### Job demands, decision latitude and social support

Data on job demands, decision latitude, and social support at the workplace were collected in 2015. The three constructs were measured with subscales of the Swedish Demand-Control-Support Questionnaire [41, 46, 47]. Answers on all three subscales were scored on a 4-point frequency scale (often; sometimes; seldom; never). We made sum scores for all three subscales by adding the item scores for each subscale.

Psychological job demands were measured with five items covering effort, time pressure and conflicts in the work setting. The job demands subscale had good internal consistency (α = 0.778). Decision latitude was measured with six items that assess the degree of control and discretion in the work setting. The job decision latitude subscale had internal consistency below the expected alpha (α = 0.597). For this reason, the results regarding this subscale need to be interpreted prudently. Social support at work was measured with six items that capture aspects of supportive relationships and atmosphere in the work setting. The social support subscale had high internal consistency (α = 0.845).

### Ethics

The study was approved by the Regional Committee for Medical and Health Research Ethics of Western Norway (REK-West, no 088.08). Written informed consent was obtained from all participants.

### Statistical analyses

Descriptive statistics for all relevant variables were calculated. A hierarchical multiple linear regression with nurses’ turnover intention in 2016 as the dependent variable was conducted. The first model included only SWD in 2015 as the independent variable. The second model also included age, sex, number of years working as a nurse, number of night shifts in previous year, shift work schedule, the three sum score variables capturing social support, job demands, and decision latitude, and dummy variables for each of the shift work categories outlined above. Preliminary analyses were conducted to ensure no violation of the assumption of normality, linearity, multicollinearity and homoscedasticity. Multicollinearity diagnostics revealed that two of the dummy variables for work schedules had VIF and tolerance values that were somewhat above and below the recommended thresholds, respectively. However, this threat is mitigated by the fact that these are dummy variables that are used for control purposes. We also ran the regression analyses without these variables and the pattern of results reported below remained the same. We therefore report the full regression analyses. All analyses were conducted using IBM SPSS Statistics 28 for Windows.

## Results

889 respondents were included in the final sample. The mean age was 39.5 years (SD = 8.6), 89.6% were female, 76.4% were living with a partner and 64.7% had children living at home. The mean number of night shifts in the preceding year was 26.8 (SD = 37.4). In total, 41.3% of nurses worked two-shift schedules, 44.8% worked three-shift schedules, 8.5% worked night shifts only, 0.1% worked evening shifts only and 5.3% worked other schedules that included night work. The numbers of observations for the different variables vary somewhat due to some missing data.

In 2015, 40.3% of the nurses were classified as having SWD. The mean turnover intention score in the subsequent year was 7.3 (SD = 3.8) and the median was 6. On the JDCS model variables, the mean scores were as follows: job demands = 14.4 (SD = 2.7), decision latitude = 12.7 (SD = 2.1), social support = 10.7 (SD = 2.9).

The results from the first model in the hierarchical linear regression showed that SWD predicted turnover intention one year later, i.e., from 2015 to 2016 (F_1,835_ = 6.00, *p* < 0.05; β = 0.084, *p* = 0.015) (Table 1). In the second model (F_8,828_ = 10.55, *p* < 0.001), the association between SWD and turnover intention remained significant (β = 0.085, *p* = 0.014) also when controlling for the other variables included in the model. Further, there was a negative association between age and turnover intention (β = -0.113, *p* = 0.002) and a negative association between workplace social support and turnover intention (β = -0.291, *p* < 0.001).

**Table 1:** Determinants of nurses’ turnover intention

	**B**	**SE**	**β**	**T**	**ΔR**^**2**^
**Step 1**					
Shift work disorder	0.660	0.267	0.086	2.473*	0.007
**Step 2**					
Shift work disorder	0.639	0.264	0.083	2.424*	0.116
Age	-0.047	0.016	-0.108	-2.878*	
Sex	-0.317	0.409	-0.026	-0.774	
Organizational tenure (# years as a nurse)	-0.034	0.034	-0.037	-0.987	
Night shift load (# night shifts last year)	0.008	0.005	0.081	1.622	
Work schedule: Only night shifts	-0.118	0.765	-0.009	-0.154	
Work schedule: Day and evening shifts	1.178	0.595	0.154	1.979	
Work schedule: Day, evening and night shifts	0.188	0.567	0.025	0.332	
Workplace social support	-0.366	0.047	-0.281	-7.727**	
Workplace job demands	-0.006	0.051	-0.004	-0.109	
Workplace decision latitude	0.078	0.062	0.044	1.262	

The table shows results from a hierarchical multiple linear regression predicting turnover intention, in which model 1 included only shift work disorder (measured one year earlier) as a predictor, while model 2 also included the additional correlates listed in Step 2. Asterisks indicate the following *p*-values: *p < .05 **p < .01. The reference category for the work schedule dummy variables is “other schedules involving night work”, cf. the method section above

## Discussion

In line with our hypothesis, the results showed that SWD was positively associated with turnover intention one year later. This result also remained significant when controlling for age, sex, organizational tenure, night shift load, work schedule, and workplace social support, demands and decision latitude. Importantly, two of the other correlates in the regression analyses, age and social support were negatively associated with turnover intention. The findings thus reveal that nurses with SWD express a stronger inclination to quit than do nurses without SWD. This intention is, also stronger among younger nurses and among those who do not experience a socially supportive work environment. While previous studies have found high turnover intention among shift workers, the present study is to our knowledge the first to show that SWD is associated with turnover intention.

Nurses with SWD may thus be more likely to seek jobs with more preferable working time arrangements, for instance day work only. Such work may alleviate the distressing symptoms of SWD. However, this interpretation relies on the assumption that nurses who experience SWD attribute the symptoms thereof to the working schedules and thus views turnover as a solution. Our data do not provide insight into such sensemaking processes. We only demonstrate the association between these phenomena and cannot draw causal conclusions about the relationship between them.

It is important to note that although there is an association between SWD and increased turnover intention, this does not necessarily lead to actual turnover. Previous studies are suggestive of a relatively weak relationship between turnover intention and actual turnover [48]. We should therefore interpret the findings on the relationship between SWD and turnover intention with caution. There is still reason to take the association between these phenomena seriously and consider possible ways to address it.

The findings that age and workplace social support were negatively associated with turnover intention are also interesting. Previous research shows that newly educated nurses, who tend to be younger, have higher turnover [49] and are more likely to quit to seek career advancement [50, 51]. Other factors, such as the question of whether experienced nurses become more embedded into the workplace and therefore have lower turnover, might also explain this association [52]. Higher age has indeed been highlighted as a protective factor against turnover and turnover intention [50]. It is an open question whether the association between turnover intention and age results from work seniority, rather than of age per se [50]. In our analyses, age rather than organizational tenure was significantly associated with turnover intention.

The finding that workplace social support was negatively associated with turnover intention might suggest that social support has a protective effect with regard to nurses’ turnover. This would imply that being in a socially supportive environment can make nurses more tolerant to adverse effects of shift work.

A central question is how the problem of SWD among nurses should be addressed. For persons with SWD, turnover can indeed be a solution, as it can make it possible to quit working shifts through other job opportunities. For nurses who wish to stay in the job, a possibility is to seek treatment for SWD. Treatment mainly comprises pharmacological interventions addressing the sleep–wake symptoms as well as chronobiological therapies aiming to realign intrinsic biological rhythms to the externally imposed shift-work schedule [53]. Other solutions put more emphasis on prevention and may as such include health promotion strategies [54], napping, sleep hygiene and education, changes in the shift work scheduling in accordance with the nurses’ preferences, as well as sufficient rest between shifts and exercise [55].

### Limitations

This study has some limitations that should be noted. The study was conducted among Norwegian nurses, of whom the vast majority were female. Furthermore, the mean age of the sample was 39.5 years, thus indicating a healthy worker effect, i.e. that turnover may have occurred at earlier ages for nurses who would therefore not be part of the current sample. While our data does not allow us to investigate this, it is likely that turnover intention would have been higher if these nurses had been included. It is also an interesting question whether SWD would have been higher in the sample of nurses who already chose to leave the job, but this is uncertain and needs to be investigated further. Consequently, the sample characteristics need to be taken into account when assessing the possibility to generalize the findings to other populations and settings. Furthermore, while the number of respondents was fairly high, it is difficult to ascertain the degree of representativeness of our sample, since we do not have data on non-participants which would have allowed for a comparison of characteristics of participants versus non-participants.

We should also note that it is a limitation that the data in our study did not allow for causal inference. Although the regression included several relevant variables, we cannot be sure if other variables would potentially affect the relationships under investigation. While the results showed that nurses who experienced low social support at work had higher turnover intention scores, our study cannot shed light on whether an increase in perceived social support (by whichever measure) would actually reduce turnover intention. It is also a limitation of our study that we do not explore moderated relationships. Future studies could explore such relationships (e.g., SWD x stress) as predictors or turnover intention. Another approach could be to conduct latent profile analysis of workers based on both demographic and work-related variables and investigate if turnover intension predicts latent profile membership [56]. Finally, we should note that the explained variance is not very high, which implies that there are unobserved predictors that may be important for the understanding of the relationships under investigation.

## Conclusion

This study showed that SWD is associated with turnover intention, even when controlling for individual and work-related variables. Age and workplace social support were negatively associated with turnover intention. These results only reveal associations and do not necessarily imply causality. Further research should investigate these relationships using other research designs that may allow for causal inferences.

## Data Availability

The datasets used in the current study are available from the corresponding author on reasonable request.
